# An assessment of antenatal care among Syrian refugees in Lebanon

**DOI:** 10.1186/s13031-015-0035-8

**Published:** 2015-02-26

**Authors:** Matthew Benage, P Gregg Greenough, Patrick Vinck, Nada Omeira, Phuong Pham

**Affiliations:** University of Missouri Medical School, Columbia, MO USA; Brigham & Women’s Hospital, Harvard Medical School, Boston, MA USA; Harvard Humanitarian Initiative, Harvard University, Cambridge, MA USA; Harvard School of Public Health, Boston, MA USA; Caritas Lebanon, Beirut, Lebanon

**Keywords:** Antenatal, Reproductive health, Family planning, Refugee, Syria, Lebanon, Contraception

## Abstract

**Background:**

After more than three years of violence in Syria, Lebanon hosts over one million Syrian refugees creating significant public health concerns. Antenatal care delivery to tens of thousands of pregnant Syrian refugee women is critical to preventing maternal and fetal mortality but is not well characterized given the multiple factors obtaining health data in a displaced population. This study describes antenatal care access, the scope of existing antenatal care, and antenatal and family planning behaviors and practice among pregnant Syrian refugees in various living conditions and multiple geographic areas of Lebanon.

**Methods:**

A field-based survey was conducted between July and October 2013 in 14 main geographic sites of refugee concentration. The assessment evaluated antenatal services among a non-randomized sample of 420 self-identified pregnant Syrian refugee women that included demographics, gestational age, living accommodation, antenatal care coverage, antenatal care content, antenatal health behaviors, antenatal health literacy, and family planning perception and practices.

**Results:**

In total, 420 pregnant Syrian refugees living in Lebanon completed the survey. Of these, 82.9% (348) received some antenatal care. Of those with at least one antenatal visit, 222 (63.8%) received care attended by a skilled professional three or more times, 111 (31.9%) 1–2 times, and 15 (4.3%) had never received skilled antenatal care. We assessed antenatal care content defined by blood pressure measurement, and urine and blood sample analyses. Of those who had received any antenatal care, only 31.2% received all three interventions, 18.2% received two out of three, 32.1% received one out of three, and 18.5% received no interventions. Only (41.2%) had an adequate diet of vitamins, minerals, and folic acid. Access, content and health behaviors varied by gestational age, type of accommodation and location in Lebanon.

**Conclusions:**

Standards of antenatal care are not being met for pregnant Syrian refugee women in Lebanon. This descriptive analysis of relative frequencies suggests reproductive health providers should focus attention on increasing antenatal care visits, particularly to third trimester and late gestational age patients and to those in less secure sheltering arrangements. With this approach they can improve care content by providing early testing and interventions per accepted guidelines designed to improve pregnancy outcomes.

## Background

Three years of violence in Syria—since March 2011—have resulted in an unprecedented level of population displacement. By May 2014, an estimated 9 million Syrians had been displaced, including 2.7 million who have crossed borders to seek refuge in Lebanon, Jordan, Turkey and Iraq. The UN High Commissioner for Refugees (UNHCR) estimates that Lebanon hosts the largest number of refugees from Syria, over 1 million people, representing more than one-fifth of Lebanon’s pre-crisis population [1]. In contravention to other massive population displacements in which refugees predominantly live in camp settings, the Lebanese government, while welcoming refugees, has formally rejected the establishment of camps. As a result, by October 2013, a majority (70%) of refugees were spending assets to rent apartments and homes or living with friends and relatives in apportioned dwellings; the remainder, with fewer resources, have been finding any place they could, including informal makeshift arrangements of tents or unused construction materials, abandoned buildings, worksites with unfinished construction, collection centers, garages, even animal sheds [2,3].

Around the world, conflict and forced displacement cause losses of lives and livelihoods, increase poverty and the risk of disease transmission, and disrupt life-sustaining services such as reproductive health, antenatal care, and family planning [4,5]. Pregnant mothers and newborn babies are particularly at risk. Women in conflict areas experience worse pregnancy outcomes, including increased fetal mortality [6], low birth weight [7], premature labor, antenatal complications and an increase in puerperal infections [8] compared to pre-conflict levels. Easily preventable maternal and neonatal deaths were among the leading causes of death for Afghan refugees in Pakistan [9]. A 2003 study found strong associations between conflicts and maternal health: 16 out of the 25 countries with the worst status of mothers’ wellbeing were experiencing conflict [10].

Evidence from ethnographic and qualitative studies suggest that conflict reduces antenatal health care utilization by inciting insecurity through intimidating health care workers and instilling fear in pregnant women seeking care [11,12]. Conflict and forced displacement also reduce access to contraceptives [13]. Women in conflict-affected and/or refugee situations tend to have little control over financial resources, less access to transport, increased domestic burden, increased gender based violence experiences, and less control over family planning decisions.

Despite the mounting evidence of the effects of conflict and displacement on reproductive health, the needs and priorities for care among refugee populations remain largely unknown, especially regarding antenatal care [14]. Antenatal health encompasses the care that is provided to a pregnant woman from conception to the initiation of labor with the goal of preventing morbidity and mortality of both the mother and the newborn [15]. Disturbance in regular antenatal care among refugees are correlated with reproductive health problems [16]. However, when camp-based registered refugee populations in host countries receive essential antenatal and obstetric care with culturally competent services capable of detecting and treating pregnancy-related complications or risks of complications, pregnancy-related outcomes come very close to those for a non-displaced population [17-19].

Among Syrian refugees in Lebanon, antenatal care represents a significant challenge affecting tens of thousands of pregnant or lactating women. A 2013 population-based assessment in Lebanon found pregnant or lactating women in 41% of the Syrian refugee households [20]. A separate 2012 study found that the main barriers to contraceptive use were high cost, distance from services, inadequate quantity, and unavailability of desired contraceptive [21]. The delivery of antenatal care to refugees is complicated by the accelerating influx of refugees and the decentralized nature of their living conditions. These two factors make it difficult for the UN Population Fund (UNFPA) and their non-governmental organization (NGO) partners to identify vulnerable populations for reproductive health programming and for UNHCR to register them, a status which entitles them to contracted government and private health facilities [22]. Because Syrian refugees in Lebanon are scattered across more than 1400 locations, antenatal care is fragmented.

Within the current health delivery system (consisting of the Lebanese Ministry of Health and humanitarian NGOs), UNHCR subsidizes a range of health services to Syrian refugees in Lebanon. In terms of antenatal care for registered pregnant women, UNHCR covers most of the fee for four antenatal care visits, 85% of laboratory costs, and 75% of delivery costs; supplements and two ultrasounds are provided free of charge. Access to breastfeeding and personal hygiene awareness sessions are also provided, as well as free access to family planning services (birth control pills, condoms and IUD insertions) and two postnatal consultations. Unregistered Syrian refugee women are eligible for one visit to a primary health care center supported by UNHCR. Privately funded primary health care centers or dispensaries that are not subsidized by UNHCR also offer health services to Syrian refugees, including ANC. The quality, number, type and prices of these services differ according to the means of each center/organization offering health care. Despite the distribution of information leaflets, information about the location, type and conditions of health services mostly circulates among the refugee community by word of mouth, leading to varying accessibility. New arrivals usually have more difficulty in accessing information and consequently healthcare.

This study was undertaken to provide a better understanding of the antenatal care needs of pregnant Syrian refugee women in Lebanon. It uses a survey designed to characterize antenatal care access, the scope of existing antenatal care, and antenatal and family planning behaviors and practice among pregnant Syrian refugees in multiple living conditions and multiple geographic areas of Lebanon.

## Methods

A field-based survey was conducted between July and October 2013 to collect information on antenatal services among a non-randomized convenience sample of 420 self-identified pregnant Syrian refugee women who visited NGO-staffed migrant health centers or community social centers located in high density refugee areas. These centers provided ANC as part of primary health services; ANC was provided care free of charge regardless of UNHCR registration status. Random sampling was not feasible given the nature of urban refugees combined with finding the study target population of pregnant Syrian refugee. A respondent’s pregnancy was validated by a follow up question to identify the method she used to determine pregnancy.

A total of 14 sites in four main geographic areas of refugee population concentration in Lebanon were selected for the study: Beirut and Mount Lebanon (Sin el Fil, Mreijeh, Taalabeya, Saint Michael, Rayfoun, Sarba, Deir el Kamar); Bekaa (Zahle, Baalbeck, Bekaanord); South Lebanon, (Saida, Tyre, Deir el Ain), and North Lebanon (Tripoli). The sites had the highest density of refugees in each of the four main geographic areas, as reported by the UNHCR. At each site, self-identified pregnant Syrian women were approached for voluntary inclusion. Participants were informed of the nature and objectives of the study, and consented to participate freely and voluntarily. No incentives were given to participants.

Interviews were conducted one-on-one, in private, at the centers or at the respondent’s home. A total of twenty-four Lebanese community health and maternal social workers working with an international humanitarian non-governmental organization (NGO) were trained to conduct the interviews. Training on the survey instrument, interviewing techniques, and research protocols on subject rights, confidentiality, and right to refusal was carried out over a two day period.

The interviewers used a modified Multiple Indicator Cluster Survey 4 (MICS 4, UNICEF) for individual women with birth history that included 51 items entailing demographic variables, living condition, antenatal care coverage, antenatal care content, antenatal health behaviors, antenatal health literacy, maternal health in Syria, and family planning perception and practices. Antenatal coverage and content followed the World Health Organization guidelines of at least four antenatal care visits and three services including blood pressure measurement, urine and blood sample analyses [23]. The survey was translated into Arabic by NGO staff and professional translators, back translated, field tested and piloted.

Non-identifiable data was stored on Microsoft Excel and analyzed with IBM SPSS 20.0 for descriptive statistics. The study was approved by the institutional review board of the University of Missouri School of Medicine. The authors did not seek IRB approval in Lebanon. Instead, the authors sought informal review for the study through a local team of experienced reproductive health and social services providers at Caritas International, a non-governmental organization working in Lebanon. These persons provided insight on the appropriateness of the protocol based on their direct experience working with the beneficiaries.

## Results

### Description of participant population

All 420 pregnant Syrian refugee women respondents residing across the four regions of Lebanon completed the survey (Table 1). Of these, 29 (6.9%) were ‘youth’ pregnancies (ages 14–18 years); 208 (49.5%) were between ages 19–29; 139 (33.1%) were ages 27–34; and 44 (10.5%) were in the high-risk older population (age 35–42 years). Participants trended towards having little education: nearly three quarters had no formal education or had completed elementary school only. The distribution of pregnancy length for the respondents was roughly equal across the three trimesters with 10.5% beyond 40 weeks gestation. Two-thirds were registered with UNHCR.

**Table 1:** **Description of participant population, n = 420**

	**All women (n = 420)**	
	**#**	**(%)**
Registered with UNHCR		
Yes	277	(66.0)
No	143	(34.0)
Age (years)		
14–18	29	(6.9)
19–26	208	(49.5)
27–34	139	(33.1)
35–42	44	(10.5)
Education		
None	63	(15.0)
Elementary	246	(58.7)
Secondary	95	(22.7)
University	15	(3.6)
Unknown	1	(0.002)
Length of pregnancy		
First trimester	121	(28.9)
Second trimester	139	(33.2)
Third trimester	114	(27.2)
>40 weeks	44	(10.5)
Don’t know	2	(0.4)
Length of time in Lebanon		
<1 month	48	(11.4)
1–2.9 months	54	(12.9)
3–5.9 months	85	(20.2)
6–11.9 months	148	(35.2)
12–17.9 months	54	(12.9)
18–23.9 months	22	(5.2)
>24 months	9	(2.1)
Type of accommodation		
Hosted/Sheltered	93	(22.1)
Rented apartment	226	(53.8)
Tent/Squatting	101	(24.1)
Geographic location		
Beirut and Mount Lebanon	154	(36.7)
Bekaa	132	(31.4)
South Lebanon	95	(22.6)
North Lebanon	39	(9.3)
	Mean	S.D.
People per accommodation	4.62	3.26
Children under 5 in Home	1.65	2.00

The population’s time in Lebanon is normally distributed with the majority between 6–12 months. Over half of the participants (53.8%) lived in rented apartments, indicative of more secure living arrangements and financial assets; 22.1% lived in UN organized collective shelters or were hosted by Lebanese families, arrangements indicative of subsidized and supportive assistance; and 24.1% lived in tents or squatted in work-related spaces (construction sites, garages or factories), the least secure environments and consistent with a concurrent lodging study [24]. The average number of people in each accommodation was 4.62 (n = 408, SD = 3.26), with the average number of children under five years being 1.65 (n = 417, SD = 2.0). The proportion of older women (aged 35 and above) living in insecure arrangements (tent/squatting) was 47.7%, twice as many as in any other age group.

### Coverage of antenatal care

The number of antenatal visits during the current pregnancy and skill level of providers were assessed (Table 2). In our study, 72 (17.1%) respondents had not accessed any antenatal care compared to 66 (15.7%) with the requisite four or more antenatal visits (Table 2). Of those with at least one antenatal visit, 222 (63.8%) received care attended by a skilled professional three or more times, 111 (31.9%) 1–2 times, and 15 (4.3%) had never received skilled antenatal care. The longer the gestational age, the more likely a woman was to have received antenatal care, and at a greater frequency. Among the 141 women in advanced gestational ages (third trimester and greater than 40 weeks), 42 (29.7%) received an adequate amount of maternal visits from skilled providers.

**Table 2:** **Antenatal care coverage among women (n = 420)**

**Number of antenatal visits**	**All gestational age (n = 420)**		**Gestational age of 27 weeks or above (n = 158)**	
	**#**	**(%)**	**#**	**(%)**
0	72	(17.1)	17	(10.7)
1	136	(32.4)	26	(16.5)
2	92	(21.9)	40	(25.3)
3	52	(12.4)	33	(20.9)
> = 4	66	(15.7)	42	(26.6)
Unknown	2	(0.01)		
Skilled antenatal care visits *only those with at least 1 antenatal visit	(n = 348)		(n = 141)	
Never	15	(4.3)	11	(7.8)
1–2 times	111	(31.9)	55	(39.0)
> = 3	222	(63.8)	75	(53.2)

Table 3 describes factors typically related to antenatal care access including UN registration, educational status, current accommodation, and regional location of respondents. Among pregnant women registered with UNHCR, only 12.6% had not received antenatal care compared to 25.9% of unregistered refugees. Of registered respondents, 30.7% had 4 or more antenatal visits compared to 24% unregistered respondents. For women who were beyond 40 weeks, 36.7% of registered women had 4 or greater visits opposed to only 18.2% of unregistered women.

**Table 3:** **Factors related to antenatal coverage, n = 420**

	**Had prior antenatal care visit?**			
	**Yes**		**No**	
	**#**	**(%)**	**#**	**(%)**
**Total (n = 420)**	**348**	**(82.9)**	**72**	**(17.1)**
Registered with UNHCR				
Yes (n = 277)	242	(87.4)	35	(12.6)
No (n = 143)	106	(74.1)	37	(25.9)
Age				
14–18	27	(93.1)	2	(6.9)
19–26	117	(85.1)	31	(14.9)
27–34	115	(82.7)	24	(17.3)
35–42	29	(65.9)	15	(34.1)
Education				
< Secondary (n = 309)	255	(82.5)	54	(17.5)
≥ Secondary (n = 110)	92	(83.6)	18	(16.4)
Type of accommodation				
Rented apartment (n = 226)	202	(89.4)	24	(10.6)
Hosted/Sheltered (n = 93)	73	(78.5)	20	(21.5)
Tent/Squatting (n = 101)	73	(79.3)	28	(20.7)
Geographic location				
Beirut and Mount Lebanon	134	(87.0)	20	(13.0)
Bekaa	99	(75.0)	33	(25.0)
South Lebanon	86	(90.5)	9	(9.5)
North Lebanon	29	(74.4)	10	(25.6)

The proportion of women having received antenatal care was lowest among the high-risk pregnant women (defined as age >35 years): 65.9% of the women aged 35 and above had prior antenatal care compared to over 80% in every other age group. Among the most educated women (those respondents that had completed secondary education and/or attended university) 13.3% of those women had not received antenatal care compared to 22.2% of the least educated (those with less than secondary education). Those living in more secure sheltering arrangements (rented apartments and/or hosted by families) were more likely to have had an antenatal visit compared to those with more tenuous living arrangements such as living in collective shelters, non-organized tents or as squatters. Regional differences were also evident: of women in Beirut and the South, less than 15% did not receive antenatal care compared with 25% of those in the Bekaa and in the North.

### Scope of antenatal care

The survey assessed whether women who had antenatal care received any of three basic services: blood pressure measurement, and urine sample and blood sample analyses. The survey also assessed the prevalence of tetanus prophylaxis. Of those pregnant women who had received any antenatal care, 31.2% received all three interventions, 18.2% received two out of three, 32.1% received one out of three, and 18.5% received no interventions (Table 4). An overwhelming majority (90.5%) had not received tetanus prophylaxis. When asked directly, 42.1% of the women said they were not told “about things to look out for that may suggest problems with the pregnancy”.

**Table 4:** **Scope of antenatal care among women with at least one visit (n = 348)**

	**#**	**(%)**
**Number of services obtained (n = 340)**		
0	63	(18.5)
1	109	(32.1)
2	62	(18.2)
3	106	(31.2)
Received tetanus prophylaxis (n = 348)		
Yes	28	(8.0)
No	315	(90.5)
Don’t know	5	(1.4)
Health awareness (n = 347)		
Yes	194	(55.9)
No	146	(42.1)
Don’t know	7	(2.0)

The proportion of respondents who received the complete basic scope of antenatal care increased with the number of visits (coverage). Table 5 shows that of those receiving care one time, 18.8% received adequate antenatal care content, while 44.6% of those respondents that had four or more visits received the three basic interventions. Regional difference plays a role in adequate antenatal care content with women in Bekaa receiving notably fewer interventions than women in the South, Beirut, or North areas, a finding corroborated by similar regional differences in pregnancy health education.

**Table 5:** **Antenatal coverage in relation to antenatal content**

	**Antenatal coverage (# visits)**							
	**1**		**2**		**3**		**> = 4**	
	**#**	**(%)**	**#**	**(%)**	**#**	**(%)**	**#**	**(%)**
Number of services obtained (n = 340)								
0	40	(30.1)	14	(15.4)	5	(9.8)	4	(6.2)
1	40	(30.1)	29	(31.9)	18	(35.3)	22	(33.8)
2	28	(21.1)	14	(15.4)	10	(19.6)	10	(15.4)
3	25	(18.8)	34	(37.4)	18	(35.3)	29	(44.6)
Region								
	Beirut n = 133		Bekaa n = 99		South n = 81		North n = 29	
	#	(%)	#	(%)	#	(%)	#	(%)
Adequate antenatal content	46	(34.6)	16	(16.2)	28	(34.6)	17	(58.6)
	Beirut n = 133		Bekaa n = 99		South n = 86		North n = 29	
	#	(%)	#	(%)	#	(%)	#	(%)
Health awareness by region (n = 347)								
Yes	88	(66.2)	37	(37.4)	57	(66.3)	12	(41.4)
No	43	(32.3)	59	(59.6)	29	(33.7)	15	(51.7)
Don’t know	2	(1.5)	3	(3.0)	0	(0)	2	(6.9)

### Antenatal health practices

Our study assessed self-reported iron intake; adequate vitamins, minerals, and folic acid intake; and smoking behaviors (Table 6). In a region with a high prevalence of iron deficiency anemia among reproductive age women, only 59.9% of women took iron tablets during their pregnancy. Even fewer (41.2%) had an adequate diet of vitamins, minerals, and folic acid. Only 9.5% of women smoked while pregnant.

**Table 6:** **Antenatal health practices**

	**Iron tablets**		**Vitamins, minerals, folic acid**	
	**(n = 410)**		**(n = 399)**	
	**# (%)**		**# (%)**	
	**Yes**	**No**	**Yes**	**No**
Region				
Beirut	105 (68.2)	49 (31.8)	78 (50.6)	76 (49.4)
Bekaa	57 (43.2)	75 (56.7)	34 (25.8)	98 (74.2)
South	65 (71.4)	26 (28.6)	45 (48.9)	47 (51.0)
North	22 (56.4)	17 (43.5)	15 (38.5)	24 (61.5)
Accommodation				
Rent	153 (69.2)	68 (30.8)	106 (48.4)	113 (51.6)
Hosted/Shelter	54 (59.3)	37 (40.7)	40 (47.6)	44 (52.4)
Tent/Squatting	42 (42.9)	56 (57.1)	26 (27.1)	70 (72.9)

Maternal health practices differed by antenatal care access, region and type of accommodation. Of those respondents that had antenatal care access, 65.4% took iron tablets compared to only 10.3% without antenatal care access. Similarly, 46.6% of women who had received antenatal care had a diet high in vitamins, minerals, and folic acid, as compared to 14.5% of women without an antenatal care visit. Slightly more women smoked-11.4% versus 9.0%--if they had not received antenatal care. Iron tablet supplementation and quality of diet was less in the Bekaa and North compared to Beirut and the South; more secure living accommodations favored iron supplementations and diet quality.

### Contraception practice and perception

**Figure 1:**
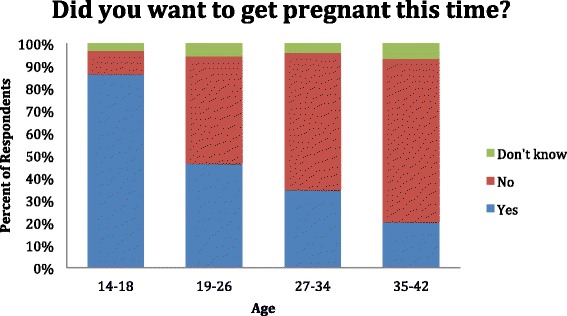
**Desire of pregnancy, by age.**

**Figure 2:**
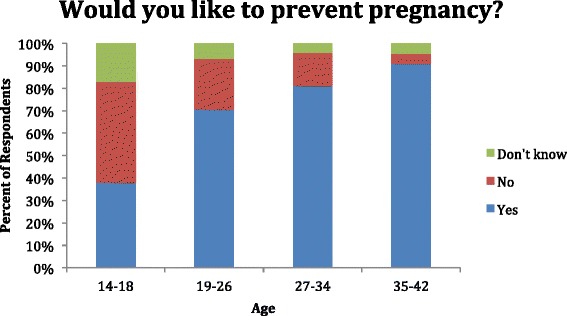
**Desire for pregnancy prevention, by age.**

Nearly three quarters of the women wished to prevent future pregnancy, and over one half (52.1%) did not desire the current pregnancy (Tables 7 and 8) (Figure 1). Despite this, 42.3% were not using any form of contraception prior to pregnancy. Women above the age of 35 desired the current pregnancy less frequently than any other group (Figure 2). Inversely, they desired pregnancy prevention more frequently than women in any other age group. For women using birth control, birth control pills followed by IUDs were preferred contraceptive choices (Table 9).

**Table 7:** **Desire of Pregnancy, by age n = 420**

**Age**	**Yes**	**No**	**Don’t know**
	**n (%)**	**n (%)**	**n (%)**
14–18	25 (86.2)	3 (10.3)	1 (3.4)
19–26	97 (46.6)	99 (47.6)	12 (5.8)
27–34	48 (34.5)	85 (61.2)	6 (4.3)
35–42	9 (20.5)	32 (72.7)	3 (6.8)
Total	179 (42.6)	219 (52.1)	22 (5.2)

**Table 8:** **Pregnancy prevention method adopted by Syrian refugee women**

	**In Syria**		**In Lebanon**	
	**(n = 414)**		**(n = 418)**	
	**# (%)**		**# (%)**	
None	141	(34.1)	177	(42.3)
Condoms	20	(4.8)	26	(6.2)
Birth control pills	139	(33.6)	98	(23.4)
IUD	88	(21.3)	72	(17.2)
Sterilization	2	(0.05)	4	(1)
Planning based on menstrual cycle	21	(5.1)	32	(7.7)
Withdrawal	2	(0.02)	4	(1)
Other	1	(0.02)	5	(1.2)

**Table 9:** **Desire for pregnancy prevention, by age n = 418**

**Age**	**Yes**	**No**	**Don’t know**
	**n (%)**	**n (%)**	**n (%)**
14–18	11 (37.9)	13 (44.8)	5 (17.2)
19–26	146 (70.5)	47 (22.7)	14 (6.8)
27–34	112 (81.2)	20 (14.5)	6 (4.3)
35–42	40 (90.9)	2 (4.5)	2 (4.5)
Total	309 (73.9)	82 (19,6)	27 (6.5)

## Discussion

This study provides, to our best knowledge, unique data on the coverage, scope and practice of antenatal care among Syrian refugees in Lebanon. The results highlight important gaps in services and practice that must be addressed. It contributes to a growing body of literature examining health care delivery–especially antenatal care delivery–to conflict affected populations.

This study finds a relatively high percentage of pregnant women reporting at least one antenatal visit (82.9%), less than percentages for Lebanon (96%) and Syria (88%) prior to the conflict [25]). This figure may be higher than expected as we did not differentiate whether the visits took place in Syria or Lebanon, where the former provides free care and the latter does not. However, the number of pregnant women reporting adequate antenatal coverage, as defined by the MICS 4 criteria, is still quite low, with only 15.7% reporting four or more visits, and only 26.6% in advanced gestational age achieving four visits. In comparison, 64% of pregnant Syrian women had at least four antenatal visits prior to the conflict [25] and the WHO reports that in low-income countries, the percentage of women that receive at least four antenatal visits ranges between 56% for rural women to 72% for urban women with an estimated 38% in the least developed countries and 50% worldwide [26].

This study finds that the percentages of pregnant women with inadequate antenatal coverage is highest among refugee women not registered with UNHCR, and among refugee women living in less secure arrangements and in areas closest to the Syrian border. (At the time of the study, there was an exponential rise in the number of refugees coming into Lebanon and nearly one-third had not been registered with UNHCR. As of January 2015, only 1% of 1.17 million refugees remain unregistered [27]). Other explanations for the inadequate level of antenatal coverage include difficulty securing transport (particularly for those living in remote locales), shortened clinic hours (one third of the study period took place during Ramadan), lack of trained health personnel, and prohibitive costs [28]. Despite subsidization for health services by UNHCR for two thirds of the sample, cost appears to be a prohibitive factor. Antenatal care services in Syria were quite cheap, and in many cases free [29], while the cost of Lebanese maternal health is more expensive [30]. With approximately 40% of all referrals to secondary and tertiary care centers being obstetric and gynecological in nature, our study indicates that pregnant women represent a significant burden of incomplete care.

The need for antenatal care is widespread among all refugee women. However, this study shows that older women (aged 35 or above) are especially vulnerable. Pregnant women in this age group had higher percentages living in more insecure living arrangements and had the least frequent percentage of visits to antenatal care services, with most visits happening late in the pregnancy. They were also the women who most frequently were willing but unable to prevent the pregnancy.

While most women are finding it possible to make at least one antenatal visit, less than one third of women are able to receive the standard of care for antenatal coverage, even in their final trimester or later. According to WHO guidelines, services are considered adequate if patients receive at the minimum blood pressure measurement and urine sample and blood sample analyses. These three interventions have been identified as necessary for detecting complications in pregnancy. While not all antenatal care has been clearly shown to limit maternal mortality, screening for pregnancy-induced hypertension, anemia, and infection, in particular, has been shown effective in detecting, treating and preventing conditions that lead to maternal mortality [31]. Immunization (especially tetanus toxoid) is also critical. Maternal and neonatal tetanus constitutes a high proportion of the total tetanus disease burden, mainly from difficulty in accessing immunizations as in the case of population displacement [32].

Predictably, increased antenatal visits improved the content of care provided to women with those receiving four or more visits having the highest levels of receiving all three interventions at 44.6%. In contrast, 18.8% of those with only one antenatal visit had all three interventions and 30.1% had none. Maternal health education was significantly lacking, especially in the border insecure areas of Bekaa and the North. With less access to health care providers, preventive messaging and early detection of complications will likely be less. Only 8.0% of women had received a tetanus vaccination, an important intervention in refugee conditions. While to our knowledge, there has not been reported tetanus cases, the recent outbreak of polio in northeast Syria and fear of reintroduction in Lebanon [33] stand as a reminder that immunizations are preferable before outbreaks, not after their arrival.

The antenatal visit should also include an education component on symptoms that portend potential pregnancy complications, and support health practices that can help circumvent poor outcomes for the mother and child. Conflicts are associated with increased food insecurity and marginalization, especially for pregnant women [34]. Maternal malnutrition is associated with increased incidence of fetal loss and adverse birth outcomes for children [35], with iron deficiency anemia being linked to increased risk for maternal mortality [36], low birth weight, lowered resistance to infection, and poor cognitive development [37].

Due to the salubrious benefit of antenatal care coverage and content on antenatal health practices, we found the expected improved antenatal health practices—iron intake, diet high in nutrients and folic acid, and less smoking—among women who had increased access to antenatal care, with the most marked differences involving the intake of iron tablets and diet high in vitamins, minerals, and folic acid between women with and without antenatal care. Smoking, although undesirable due to the deleterious effects on the mother and fetus, is well below smoking levels observed in other Middle Eastern and Northern Africa countries (9.5% vs 28.8%) [38].

Proximity to the insecure border areas of the Bekaa and North Lebanon and living in more tenuous shelter was associated with less iron and less adequate dietary intake. There was an iron and dietary decline across shelter security, with those in more stable arrangements (able to rent apartments) better than those in hosted settings and shelters and in turn better than those in tents and squatter communities.

Access to family planning, including modern contraception, empowers refugees, particularly women, to make important decisions about their reproductive health. Family planning could prevent up to 30% of the approximately 287,000 global maternal deaths that occur per year by enabling women to delay their first pregnancy and to space pregnancies at safe intervals. If successive children were born three years apart, an additional 1.6 million children under the age of five would survive [39]. The focus on family planning needs for refugee women is critical.

Less than one half the women surveyed desired their current pregnancy—with a clear differential of less preference by increasing age—and nearly three fourths sought to prevent a future pregnancy, suggesting a desire for personal agency in family planning, again with a higher preference in older women. Despite this, our study suggests displacement and forced migration to Lebanon has resulted in less contraception use, especially less use of favored methods (oral contraceptives and IUDs). This finding coupled with an increase in the use of non clinical methods for contraception such as condoms and planning based on menstrual cycle suggest increased difficulty in locating and utilizing effective forms of contraception. A mixed methods study found that barriers to contraceptive use were high cost, transport distance, inadequate number of contraceptives, and unavailability of preferred type of contraceptive [40]. While our study aligns on access challenges with this one—undertaken one year earlier—it is not clear why the two studies differed on preferred contraception use.

Although our study did not explore the reasons for pregnancy or the factors contributing to a majority of undesired pregnancies, there has been a documented increase in the number of child marriages among Syrian refugees in Jordan [41]. Poverty within the family unit, risk of sexual violence, and insecurity all play a role in this practice. Young maternal age is associated with pregnancy complications, low birth weight, preterm labor, and inadequate antenatal care [42-44].

### Limitations

Limitations of this study are those similar to other quantitative non-randomized study designs, namely the inability to make statistical inferences to the population of pregnant Syrian refugee women in Lebanon. It is not possible to quantify the weighted effect of variables with this study: lack of access to antenatal care will likely be highly associated with geographic insecurity and socioeconomic capacity but the study design can only provide descriptive percentages of those interviewed in the study. Also, convenience samples are inherently biased as respondents self-select for inclusion; many of those recruited were already seeking or intent on seeking antenatal care. The studied population is not representative of the entire Syrian pregnant refugee population since participants included were those able to access migrant centers. Also, we were unable to include the Akkar district of North Lebanon, an area of high density refugee settlement. Such insecure areas tend to have higher pockets of poverty, which could also limit access to services. Data on antenatal testing was based on a respondent’s self-report and not able to be independently verified. Based on the responses, the respondents seem knowledgeable about antenatal screening tests. However, if respondents were not fully aware of the types of testing, this could result in under-reporting. Self-reported behaviors are fraught with under-reporting and over-reporting error as respondents may either aim for some benefit (in the case of the former) or state what they think researchers may want to hear (in the case of the latter).

That said, there is value to having some descriptive understanding of the maternal and reproductive health needs of a hard-to-reach but vulnerable population of interest to the humanitarian community.

## Recommendations and conclusions

Standards of antenatal care are not being met for pregnant Syrian refugee women in Lebanon, despite UNCHR registration status. The current state risks compromising the health of thousands of women and their unborn children. The percentages here suggest that maternal and reproductive health providers in Lebanon and within the humanitarian community should focus attention on increasing antenatal care visits, particularly to third trimester and late gestational age patients and to those in less secure sheltering arrangements, and in such a way as to increase the likelihood of multiple visits.

The health care delivery system is working above capacity in Lebanon. Initiatives that reinforce the capacities of primary health care and social development service and enhance refugee access to these centers are critical. A geographic gap analysis that identifies distributions of centers with population would guide program deployments. Similarly a study with a geographic focus that probes the underlying reasons for insufficient access to antenatal care and family planning, and the reasons behind unwanted pregnancies, would customize interventions. (Fortunately, a UNHCR-funded research study on prenatal and maternal health access in Akkar is currently underway). Incentives and communication strategies that foster UNHCR registration would ameliorate the cost barriers to access and allow household resources to be used for other vital needs.

Once access is secured, maintaining consistent records, ensuring supply chains and access to contraception and family planning, adhering to antenatal care guidelines, and intervening and referring in a timely and efficient manner to higher levels of care would improve maternal and neonatal health outcomes. This study identified a need to focus on older pregnant women in particular. WIth this approach, the humanitarian health NGOs and the UN humanitarian field operations, working with the Lebanese Ministry of Health, can improve care content by providing testing early in the series of visits per well-established and accepted guidelines and facilitate cost-effective timely and practical interventions known to improve pregnancy outcomes.

## Abbreviations

WHO: World health organization
IAWG: Interagency working group
MICP: Minimum initial service package (misp) for reproductive health in crisis situations
UNHCR: United nations high commissioner for refugees
UNFPA: United nations population fund
NGO: Non-governmental organization
MICS 4: Multiple indicator cluster survey
UNICEF: United nations children’s fund
